# Changing Parental Knowledge and Treatment Acceptance for ADHD: A Pilot Study

**DOI:** 10.1177/00099228221124676

**Published:** 2022-09-28

**Authors:** J. F. Dixon, R. Akins, E. Miller, J. Breslau, S. Gill, E. Bisi, J. B. Schweitzer

**Affiliations:** 1 Department of Psychiatry and Behavioral Sciences, University of California, Davis, Sacramento, CA, USA; 2 UC Davis MIND Institute, School of Medicine, University of California, Davis, Sacramento, CA, USA; 3 Department of Pediatrics, University of California, Davis, Sacramento, CA, USA; 4 Department of Pediatrics, University of Pittsburgh, Pittsburgh, PA, USA; 5 Rand Corporation, Pittsburgh, PA, USA; 6 Palo Alto University, Palo Alto, CA, USA

**Keywords:** ADHD knowledge, parenting, treatment acceptance and utilization

## Abstract

This pilot study assessed the feasibility and potential effectiveness of a single-session workshop in modifying parental beliefs/knowledge about attention-deficit/hyperactivity disorder (ADHD) in children and impact on treatment acceptance/utilization. Concerns raised by school professionals about lack of treatment follow-through after ADHD diagnosis and parental misinformation about medication usage catalyzed this project. A single-group pre-post quasi-experimental design was used. Sixty-eight parents completed ADHD knowledge/belief scales and stress inventories, and pre-ADHD and post-ADHD information workshop. Follow-up calls were made after the workshop to assess treatment utilization. Parents/caregivers experienced significant knowledge and belief changes regarding medication efficacy, willingness to accept physician treatment recommendations, and rejection of non-empirically based treatments. Follow-up data showed that 41% of contacted participants met with physicians to discuss medication utilization and behavioral treatments. Brief, one-session psycho-educational workshops were feasible and impacted parental beliefs and behaviors regarding scientifically supported interventions for ADHD.

Attention-deficit/hyperactivity disorder (ADHD) is a neurodevelopmental disorder characterized predominantly by symptoms of inattention, impulsivity, and hyperactivity.^1-3^ Children diagnosed with ADHD experience significant impairment in academic and social functioning.^4^ Attention-deficit/hyperactivity disorder is the most commonly diagnosed child and adolescent psychiatric disorder,^2,5^ with a 5% to 7% prevalence rate among youths.^6^

Research supports the use of both pharmacological and behavioral treatments for ADHD.^7,8^ Although the use of stimulant medications for the treatment of ADHD is well established,^9,10^ acceptance of and adherence to stimulant treatment remain low,^11^ with nonadherence to treatment ranging from 13% to 64%.^12^

When asked about their perspective on the decision to initiate medication treatment for ADHD, parents describe ambivalent feelings regarding the decision to use stimulants with their children, as well as fears of side effects, lack of family support for the decision to medicate, and guilt specific to social pressure about medicating children.^13^ Approximately half of youths diagnosed with ADHD do not begin stimulant treatment, and of those who do, roughly half discontinue it within a year.^14^ Furthermore, hesitation among parents regarding medication usage has been attributed to information presented in the media. In addition, some parents believe that too many children receive medication for ADHD,^15^ despite evidence to the contrary.^16^ This ambivalence persists in the face of strong evidence supporting the efficacy of stimulant medications in the short-term treatment of the core symptoms of ADHD.^12,17,18^

The negative attitudes held by many parents about the acceptability of medication as part of an optimal course of treatment likely impact both treatment-seeking and adherence behavior.^19,20^ Underscoring the need to bridge negative parental views and treatment acceptance is essential as this combination can result in untreated ADHD.^13^ Although parents often express a preference for behavioral treatments, adherence rates for behavioral therapies are also low due to issues including the time commitment required or difficulty accessing services.^21^

An examination of parental treatment preferences found^22^ that “shared decision-making” (SDM), in which parental preference and goals are assessed as part of the clinical evaluation, led to higher treatment initiation of both pharmacological and behavioral treatments. Although findings suggest that parents trust information presented to them by their child’s physician,^23^ their concerns about treatment may not be elicited in a way that keeps them engaged in the treatment process.^21^

Previous research demonstrated a relationship between parenting perceptions, practices, and increased familial stress.^24^ Factors that influence parental stress include the parents’ awareness of psychological, academic, emotional, and behavioral problems in ADHD.^19^ In addition, parental stress has been linked to willingness for acceptance of and adherence to child ADHD treatment.^25^ Through parent education, perceptions about treatment may be modified to facilitate wider treatment acceptance and engagement.

This pilot study sought to assess the feasibility and potential effectiveness of a single-session workshop in modifying parental beliefs and knowledge about the characteristics, diagnosis, and treatment of ADHD in children. A secondary aim was to assess whether these changes in knowledge and beliefs lead to any actual changes in treatment engagement or use. A school of medicine, university-based ADHD research program partnered with a local city school system to identify relevant issues to target. A community-based participatory approach was taken in this project^26^ with the objectives identified and informed by interviews with local teachers and school administrators who indicated that parents needed increased and current information about treatments for ADHD. School personnel indicated that they felt parental misinformation regarding ADHD, their recommendations for evaluation, and potential treatment for ADHD or follow-through with already prescribed medication was hampering their student’s functioning in the school setting. This project assessed changes in parental knowledge, attitudes, and beliefs about ADHD and gathered data about changes in treatment utilization subsequent to the intervention.

## Method

### Participants

A total of 68 parents and primary caregivers participated in this study. (Note the term “parent” is used to encompass all caregiving relationships.) Study inclusion criterion was parental interest in learning more about ADHD for a child who was attending the local, city school district; there were no exclusion criteria. Study participants included parents of children considered at-risk of ADHD via parent report or who had already been diagnosed with ADHD. The children ranged in age from 4 to 15 years, of whom 50 children (40 men; 10 women) already had been diagnosed with ADHD. Of those, 50% had been prescribed medication and/or tried a behavioral therapy intervention. The local school system invited families of elementary and middle school children in the district by posting flyers and leaving telephone messages to alert them to the workshop opportunity. Childcare was provided for the children of participants. The university Institutional Review Board (IRB) approved the study, and the researchers obtained informed consent from participants at the beginning of each workshop. Caregivers were given $30 for their time.

### Design and Workshop Procedure

The study employed a single-group, quasi-experimental design. The phases of this study included a Pre-Workshop Assessment, Workshop Presentation, immediate Post-Workshop Assessment, and Follow-up Assessment to assess changes in treatment engagement or utilization. Parents attended 1 of 4 two-hour workshops, held over a 4-month period using a standardized protocol format to ensure consistency between workshops. Workshop content included information on occurrence rates, diagnostic criteria and state-of-the-art evaluation procedures, myths, treatment approaches, and evidence to support different treatments for ADHD. The presenters each gave their respective talk using a formal slide presentation followed by a question-and-answer format for each topic. Presenters at each workshop included the same 2 PhD-level clinical psychologists and a developmental and behavioral pediatrician with expertise in ADHD. Suggestions were given to parents on how to prepare for evaluations of their children. Information on the effect of the workshop on parents’ attempts to engage in subsequent services for their children was gathered. To facilitate community participation, the workshops were held in sites easily recognizable to the parents including the school district office (3 workshops) and a university site engaged in participatory work, adjacent to the community (1 workshop). Follow-up information from participants employing standardized phone interviews was gathered 12 weeks to 17 months (median approximately 10 months) following the final workshop to assess for any changes in diagnostic status, professional consultation, or treatment utilization. Initial attempts were made to contact the participants within 3 to 4 months after the workshops. However, nonresponse of the participants proved challenging, and thus, we continued to attempt follow-up data well beyond the planned period if the participant did not initially respond to increase the sample size and data on service use. The follow-up data were deidentified due to IRB constraints, such that pretest/posttest questionnaire data were not linked to follow-up with specific subjects.

### Measures

We collected demographic information including age of child, parental age, marital status, level of education, race and/or ethnicity, and previous usage of ADHD treatments (if any). At the commencement of each workshop, caregivers completed the Parenting Stress Index (PSI)–Short Form.^27^ The PSI^27^ assessed relative stress via parent self-report assessment. The short version of the scale contains 36 items and is composed of 3 subscales: Parental Distress, Difficult Child Characteristics, and Dysfunctional Parent-Child Interaction. A total summary stress scale was also calculated. The internal consistency for the PSI is 0.85.^7^ This measure allowed us to look at the relationship between parenting stress and shift in ADHD beliefs.

Parents also completed the standardized ADHD Beliefs and Attitudes Scale^28^ before and after the workshop. It consists of 27 items rated on a Likert scale from 1 to 7 (1 = disagree, 4 = neutral, 7 = agree), which together identify knowledge and opinions about the possible causes of ADHD, characteristics of children with ADHD, and treatments of the disorder. The Johnston et al^28^ scale was used to determine a baseline of current knowledge about ADHD, beliefs and treatment options, modality acceptance, satisfaction with the current treatment regimen, and parental stressors related to child management. The scale is composed of 4 subscales: Beliefs in Behavior Management, Beliefs in Medication, Beliefs in Psychological Causes/Treatments, and Beliefs in Diet/Vitamin Treatments. In preliminary findings, the internal consistency for Beliefs in Behavior Management was 0.73 and for Beliefs in Medication 0.77.^28^ Changes in this measure served as the primary outcome variable.

As noted above, for the follow-up data collection, a standardized phone interview assessed for changes in diagnostic status and whether parents sought professional consultation for ADHD and whether their child began treatment for the disorder.

### Data Analysis

All analyses were computed using SPSS software (statistics software, version 21.0) for the 3 factors from the PSI and the 4 factors from the ADHD Beliefs and Attitudes Scale. The significance threshold was set at *P* < .05 (2-tailed). If a factor showed a significant pre-workshop to post-workshop change, we performed an exploratory analysis to determine whether this change was being driven by one or more of the individual items making up the factor. For the PSI, means, standard deviations, and standard error were calculated to better assess summary scores across each of the subscales (PD = Parental Distress; PCDI = Parent-Child Dysfunctional Interaction; DC= Difficult Child; and TS = Total Stress, a combination of PD, PCDI, and DC). Finally, Pearson correlation coefficients were used to examine relationships between the ADHD Beliefs Scale and the PSI. Dependent-samples *t* test mean and standard deviation values were used to compare the changes from pre-workshop to post-workshop across each major subscale of the ADHD Beliefs and Attitudes Scale. Furthermore, to compare item-specific changes from pre-workshop and post-workshop ratings by parents in each of the major subscales of the ADHD Beliefs and Attitudes Scale, we employed a Wilcoxon signed-rank test using negative ranks. The strength of the relationship between these 2 scales was also assessed with the aim of identifying any potential relationships that could lead to testable hypotheses for future studies.

We performed Wilcoxon signed-rank test analyses for individual item responses of the ADHD Beliefs and Attitudes Scale as well as for subscale scores on the ADHD Beliefs and Attitudes Scale and PSI. Subscale scores reported here represent average item responses: items from the ADHD Beliefs and Attitudes Scale were rated on a Likert scale from 1 to 7 (1 = disagree, 4 = neutral, 7 = agree). Items from the PSI were presented as percentile scores. Listwise deletion was used to handle missing data.

Data derived from the follow-up phone interview were categorized and summed.

## Results

Fifty-five parents (81%) completed both pre-workshop and post-workshop questionnaires to assess beliefs and knowledge about ADHD. Thirteen subjects were ineligible because they failed to complete the pre-workshop and post-workshop questionnaires. Fifty-eight parents (85%) completed the PSI. Thirty-seven (51%) participants provided post-workshop follow-up data. Mean grade of ADHD diagnosis for children was second grade. The parents were well educated (an average of 14 years of education) and most were in a “Married /Remarried /Partnered” relationship (see Table 1 for full demographic information). The participant group represented racial and ethnic diversity consistent with our geographic region (72% white, 19% Hispanic, 13% African American/Black, or Mixed racial heritage) with the exception of Asian Americans who were underrepresented in this sample (3.4%).

**Table 1.** Summary of Demographic Information.

Child gender (n = 50)	Male	Female	Unreported		
Percent	64.7	26.5	8.8		
Attendee relationship (n = 68)	Mother	Father/step-father	Grandparent/great grandparent	Teacher/staff	Unreported
Percent	80.9	4.4	10.3	2.9	1.5
Parent relat. status (n = 58)	Married/remarried/partnered	Divorced/separated/widowed	Single	“Other”	
Percent	70.7	13.8	13.8	1.7	
Parent ed. level (n = 58)	Mother	Father			
M Years	15.5	14.9			
SD Years	3.0	2.9			
Parent ethnicity (n = 58)	Not Latino/Hisp	Latino/Hisp	“Other”		
Percent	70.7	19.0	10.3		
Parent race (n = 58)	Native American	Asian	African American	White	“Unknown”
Percent	3.4	3.4	13.8	72.4	6.9

The total number of respondents was 68, however, not all completed each item. Sample sizes per item indicated above.

Abbreviations: Parent relat status, parent relationship status; Parent ed. level, parent educational level, in years.

Two factors from the ADHD Beliefs and Attitudes Scale showed a significant change. These included the Beliefs in Medication factor and Beliefs in Diet/Vitamin Treatments (see Table 2). Movement on 3 items that make up these factors represents this shift. Prior to the workshops, parents endorsed beliefs that medications were safe, worked best in consort with behavioral management, and helped to alter brain chemistry. These ratings were sustained in the post-workshop assessment. However, for item 11—“Medication is almost always an effective treatment for ADHD”—the amount of change from pre-workshop (*disagree*) to post-workshop (*agree*) for this item was significant (*z* = −5.08, *P* < .001). Similarly, item 23—“I would not hesitate to medicate my child if a doctor recommended it”—also shifted from *disagree* to *agree* post-workshop (*z* = −4.19, *P* < .001). Item 19—“Vitamin therapy is useful in treating ADHD”—was part of the Beliefs in Diet/Vitamin Treatments subscale and shifted toward a belief that this is not an effective form of treatment (*z* = −4.39, *P* < .001). Following the workshop, parents were more likely to see medication as effective, less hesitant to explore medication treatment, and less likely to believe that vitamin therapy is effective. These findings are particularly important, as concern about medication implementation was an impetus for this study.

**Table 2.** Change From Pre-Workshop to Post-Workshop for Each Average Subscale Item Score of the ADHD Beliefs Scale.

Subscale	*t*	*P*	Pre-workshop		Post-workshop		Cohen’s *d*
			Mean	SD	Mean	SD	
Behavior management	−1.82	.07	6.04	0.71	6.20	0.75	0.22
Medication	−7.28	<.001*	4.46	0.95	5.22	1.13	0.73
Psychological causes	1.70	.10	2.82	0.93	2.68	0.77	−0.16
Diet	6.14	<.001*	3.96	1.22	3.32	1.38	−0.49

All items were rated on a Likert scale from 1 to 7 (1 = *disagree*, 4 = *neutral*, 7 = *agree*).

Abbreviation: ADHD, attention-deficit/hyperactivity disorder.

* Significant at the *P* < .01 level.

Consistent with reports in the literature,^24^ participants in this sample uniformly reported moderate to high levels of parenting stress. The mean for Parental Distress was at the 53rd percentile, Parent-Child Dysfunctional Interaction was at the 79th percentile, Difficult Child was at the 81st percentile, and Total Stress was at the 77th percentile. Correlations were performed (using arcsine-transformed percentile data) to explore the relationship between change in beliefs and parental stress. The results did not reveal any significant correlations between belief change and total parenting stress. However, there was a significant correlation between changes in beliefs of Behavioral Management and pre-workshop levels of Parental Distress such that higher levels of parental distress were associated with larger positive changes in beliefs about the effectiveness of behavioral management (*r =* 0.28, *P =* .04).

### Treatment Use Follow-up

The original study design called for participants to be contacted 12 weeks following workshop attendance by phone to assess what changes, if any, they made about their child’s ADHD diagnosis and treatment. However, subjects proved difficult to reach (and return phone calls) and the length of follow-up stretched to 17 months in an attempt to interview as many participants as possible. Thirty-seven participants (55.2%) provided follow-up data. (Nineteen follow-up interviews were completed 6 months post-workshop and 18 were reached 14-17 months post-workshop. An average of 3-4 contacts were needed to reach participants.) Of the contacted participants, 28 (75.7%) already had a diagnosis of ADHD at the beginning of the study. Twenty-three of the 28 were already receiving medication. Of the 5 children not already on medication, 2 families subsequently obtained a medication consultation and began medication following the training group.

There were 9 contacted participants (24.3%) for whom the family suspected ADHD, but they did not have a formal diagnosis at the beginning of the training workshop. Of these, 5 subsequently obtained evaluations and received a positive diagnosis of ADHD.

Following the parent education workshop, 15 participants (40.5%) consulted a family physician or pediatrician. Of these, 13 met to discuss medication issues specifically. Seven participants began taking medication following this consult, 1 discontinued, 1 switched medication, and 4 families met with their physician but did not make medication changes. Twelve families sought out behavioral interventions such as child-focused individual therapy or behavioral family therapies. This is an important finding as this represents 32% of our participant subjects and follows a single workshop. In the general ADHD population, only about 30% to 45% of children become engaged in any behavioral intervention.^29^

## Discussion

The results suggest that a single workshop session delivering current, evidence-based information about ADHD to a culturally and ethnically diverse parent group can modify parental beliefs to align with state-of-the-art evidence on ADHD. Results indicated that our parent group was already knowledgeable about the course and treatment of ADHD as was reflected in their overall endorsement of appropriate behavioral interventions and medication treatment. They understood the psychological underpinnings of ADHD and the limitations of dietary interventions. However, areas where parents’ beliefs shifted following workshop training included beliefs about the efficacy of medication treatment and their willingness to use this as a treatment strategy, as well as their willingness to accept a treating physician’s recommendations about treatment. Immediately following the workshop, parents had more positive beliefs regarding medication interventions. Post-workshop parent reports shifted in that they were less likely to endorse diet and vitamin therapies as effective. This is particularly important as parents often seek nutrition-based treatments because they see these as potentially less harmful than pharmacological agents.^30,31^ These findings support the conclusion that a single workshop can improve the ability of parents to participate more fully in their child’s treatment by initiating discussions with their physician because they have evidence-based data to draw upon. The ultimate hope is that parents will become better potential partners for shared decision-making with physicians. In addition, although our parent participants reported moderate to high levels of parenting stress, this did not seem to impair their ability to shift their beliefs, again suggesting that accurate and well-presented material can be delivered across a range of diverse families. Twelve out of 37 families indicated that they sought out behavioral interventions following the workshop, suggesting that parents may have become more aware of the availability of behavioral interventions through the workshop. Notably, there were frequent questions about where to obtain these services in the question-and-answer phase of the workshop, and the facilitators were able to provide suggestions for obtaining these services. However, without the presence of a control group, this is difficult to ascertain.

The literature states that efficacy for all treatments is determined to a large extent by treatment adherence.^13^ One of the first steps in treatment efficacy is the willingness to engage directly with a treating professional and to accept the recommendation to initiate a course of treatment. Of our sampled families, a substantial portion of the families we reached initiated consultation visits with their physician to discuss their child’s treatment following workshop participation. Such actions bring us closer to the optimal goal of greater treatment engagement. Notably, several parents commented at the end of the workshop that they had not felt comfortable asking their physicians for further information about treatment, but that they would now, having participated in the workshop. Parental perception that their child’s physician would not welcome questions about treatment for ADHD is critical for physicians to recognize as this may lead to lower adherence to treatment recommendations. Therefore, it is also important for physicians to discuss shared decision-making so that treatment recommendations might be initiated earlier.

Several limitations of this pilot study should be noted. While this single-session, pre-post assessment of a workshop model served as an efficient method of information dissemination, there were limitations in the design and scope of the approach. Future studies can build upon our preliminary work by testing the effectiveness of a single-session workshop by using a randomized design with comparisons between an active educational session versus a control group receiving no education. Adding a more interactive, participatory, group discussion format may also lead to higher behavioral change than just receiving standard educational information. In the future, it would also be important to assess other contextual barriers to treatment engagement (transportation, childcare, insurance, etc). Potential selection bias related to our sample is also a significant concern. Parents who elected to attend a session led by clinicians may already have some commitment to learning about efficacious treatments and approaches to ADHD. The parents were well educated. Our group expressed a desire to engage in treatment and appeared to take medical advice seriously. Strengths of this study included use of a community-participation approach, such as selection of topics identified as important by teachers and school staff; using a workshop format that was reasonable for busy families (ie, a single session, providing childcare, using well-known community spaces); and workshop facilitators representative of both psycho-educational and medical profession.

Our success with contacting only about 50% of our original participants is a limitation of the study. Recent studies indicate maintaining research participant engagement is challenging, particularly for research in behavioral health.^32,33^ A review of the literature^32^ showed that multiple attempts are required to keep participants engaged, with some studies showing averages of 8 contact attempts, and that this is not unusual. Reconnecting with busy parents of children with behavioral disorders is a particular concern. The participants with whom we were able to engage in the follow-up period may not be representative of the larger group, that is, they may demonstrate nonrandom attrition. Many of the contacted families indicated that they were using medication as a part of their child’s treatment regimen. Parents who did not make this choice are clearly underrepresented in this sample. It is possible that parents who were experiencing greater difficulty managing their child’s ADHD and greater stress may have greater challenges responding to the follow-up calls as they may have had other priorities.^32,33^ The length of time for follow-up with some families was lengthy, and there may be fundamental differences between families we were able to reach at 12 weeks and those we did not reach until 17 months. Because follow-up data were deidentified, we were not able to distinguish what group differences may have been present in the sample we were unable to reach. To increase parent response to follow-up interviews, we recommend that future studies reimburse parents for their time and participation in efforts to obtain follow-up data. Future research should systematically evaluate methods for maintaining engagement in studies, including testing different types of social networking communications.

A strength of our project is its inclusion of a diverse sample of parents, although with a sample size insufficient to explore the relationship between intervention and diversity. Future research should explore whether the effects of psycho-educational workshops are moderated by socioeconomic status and ethnic group membership on treatment participation. In summary, this study moves us closer to understanding what factors may contribute to a parent’s willingness to engage in treatment for ADHD. It demonstrates the potential of a group format for modifying parental beliefs about issues school professionals consider a high priority. Notably, knowledge gained through the workshop increased willingness of parents to partner with treating professionals.

## Author Contributions

J.F. Dixon took responsibility for supervision of all aspects of the study and wrote the first draft of the manuscript. J.B. Sweitzer and R. Akins facilitated workshops and reviewed and revised manuscript. E. Bisi participated in the literature review and reviewed and revised manuscript. S. Gill scored the study measures and reviewed and revised manscript. E. Miller and J. Breslau reviewed and revised manuscript.
